# The putative tumor suppressor gene *EphA3* fails to demonstrate a crucial role in murine lung tumorigenesis or morphogenesis

**DOI:** 10.1242/dmm.019257

**Published:** 2015-02-20

**Authors:** Jenni Lahtela, Barun Pradhan, Katja Närhi, Annabrita Hemmes, Merja Särkioja, Panu E. Kovanen, Arthur Brown, Emmy W. Verschuren

**Affiliations:** 1 Institute for Molecular Medicine Finland (FIMM), University of Helsinki, Helsinki FI-00014, Finland.; 2 Department of Pathology, HUSLAB and Haartman Institute, Helsinki University Central Hospital and University of Helsinki FI-00014, Finland.; 3 Spinal Cord Injury Team, Robarts Research Institute, University of Western Ontario, London, ON N6A 5K8, Canada.

**Keywords:** *EPHA3*, EPH receptor A3, GEMM, Adenocarcinoma, Lung morphogenesis

## Abstract

Treatment of non-small cell lung cancer (NSCLC) is based on histological analysis and molecular profiling of targetable driver oncogenes. Therapeutic responses are further defined by the landscape of passenger mutations, or loss of tumor suppressor genes. We report here a thorough study to address the physiological role of the putative lung cancer tumor suppressor EPH receptor A3 (*EPHA3*), a gene that is frequently mutated in human lung adenocarcinomas. Our data shows that homozygous or heterozygous loss of *EphA3* does not alter the progression of murine adenocarcinomas that result from *Kras* mutation or loss of *Trp53*, and we detected negligible postnatal expression of *EphA3* in adult wild-type lungs. Yet, *EphA3* was expressed in the distal mesenchyme of developing mouse lungs, neighboring the epithelial expression of its *Efna1* ligand; this is consistent with the known roles of EPH receptors in embryonic development. However, the partial loss of *EphA3* leads only to subtle changes in epithelial *Nkx2-1*, endothelial *Cd31* and mesenchymal *Fgf10* RNA expression levels, and no macroscopic phenotypic effects on lung epithelial branching, mesenchymal cell proliferation, or abundance and localization of CD31-positive endothelia. The lack of a discernible lung phenotype in *EphA3*-null mice might indicate lack of an overt role for EPHA3 in the murine lung, or imply functional redundancy between EPHA receptors. Our study shows how biological complexity can challenge *in vivo* functional validation of mutations identified in sequencing efforts, and provides an incentive for the design of knock-in or conditional models to assign the role of *EPHA3* mutation during lung tumorigenesis.

## INTRODUCTION

Lung cancer is a leading cause of cancer-related deaths worldwide. More than 85% of all lung cancers are classified as non-small cell lung cancer (NSCLC), which is further sub-classified as adenocarcinoma (ADC; ~50%) and squamous cell carcinoma (SCC; ~40%) (Chen et al., 2014). In recent years, excellent progress in molecular profiling of NSCLC has identified stratified patient groups that benefit from targeted therapies (Oxnard et al., 2013). Specifically, erlotinib or gefitinib are prescribed to patients that carry mutations in epidermal growth factor receptor (*EGFR*), and crizotinib to carriers of anaplastic lymphoma kinase (*ALK*) gene rearrangements. However, despite an increase in progression-free survival, the overall survival benefit of such tyrosine kinase inhibitors remains marginal, and profound intra- and inter-tumor genetic heterogeneity confounds effective long-term responses (de Bruin et al., 2014).

Next-generation sequencing of lung cancer patient tumors has identified numerous putative new cancer drivers, including EPH (also defined as erythropoietin-producing hepatocellular) receptor A3 (*EPHA3*), which is mutated in 6–16% of lung ADC samples (Cancer Genome Atlas Research Network, 2014; Ding et al., 2008; Imielinski et al., 2012). The EPH receptors make up the largest family of receptor tyrosine kinases (RTKs) and, together with their ephrin ligands, they control a variety of biological processes. They are classified into two subclasses based on sequence homologies, namely EPHA and EPHB receptors and their ephrin-A and ephrin-B ligands. Interaction between the EPH receptors and their ligands at cell-cell contacts triggers signaling into both the receptor- and ligand-expressing cell. Such bidirectional signaling induces changes in the actin cytoskeleton, cell-substrate adhesion, intercellular junctions and cell shape, impinging on cell movement and tissue patterning (Pasquale, 2010). Context-dependent cellular responses are finely tuned by the abundance and type of receptor-ligand pairs expressed in neighboring cells, leading to specialized cell functions known to control synaptic plasticity, insulin secretion, epithelial homeostasis and inflammatory immune responses (Gucciardo et al., 2014; Pasquale, 2010).

The expression pattern of *EphA3* in mammalian tissues suggests that there is a role for EPHA3 in neuronal development and formation of mesoderm-derived tissues (Kilpatrick et al., 1996; Kudo et al., 2005; Yue et al., 1999). However, in contrast to predictions made based on its expression in the developing medial motor column, constitutive loss of murine *EphA3* does not lead to abnormal motor axon topography (Vaidya et al., 2003). Instead, 75% of the null mice die at birth owing to cardiac abnormalities caused by defective endothelial-to-mesenchymal transition, a specific form of mesenchymal conversion that generates progenitors of the atrioventricular valves (Stephen et al., 2007).

With respect to its putative role in tumorigenesis, previous studies have indicated that EPHA3 can signal both in a kinase-dependent and kinase-independent manner, inducing both tumor-promoting and tumor-suppressing effects (Boyd et al., 2014). For example, in glioblastoma multiforme, EPHA3 is highly expressed in undifferentiated mesenchymal cells where it has been shown to confer a kinase-independent oncogenic role through regulating mitogen-activated protein kinase (MAPK) signaling (Day et al., 2013). A tumor-suppressive role of EPHA3, in particular for lung cancer, is supported by the reduction in receptor activity conferred by the point mutations found in cancers, and ligand- and EPHA3-dependent apoptosis of tumor and stroma cells upon receptor agonist treatment, suggesting that wild-type EPHA3 has anti-tumorigenic properties (Lahtela et al., 2013; Lisabeth et al., 2012; Vail et al., 2014; Zhuang et al., 2012). Furthermore, the finding that senescence elicited by acute EPHA3 loss is rescued by loss of p16^INK4A^ (encoded by *Cdkn2a*) or p53 (encoded by *Trp53*) suggests that EPHA3 mutation might promote tumorigenesis only in the absence of senescence-inducing pathways (Lahtela et al., 2013). Given the opposing outcomes of aberrant EPH-ephrin signaling, careful dissection of the tissue and cell-context-specific EPH receptor functions requires studies that utilize valid *in vivo* model systems.

**TRANSLATIONAL IMPACT**

**Clinical issue**

Lung cancer is the leading cause of cancer-related deaths worldwide. Molecular profiling to identify targetable driver mutations is increasingly being applied in the clinic, and can stratify patient groups. However, pronounced patient- and tumor-specific lung tumor heterogeneity confounds long-term or predictable clinical responses. Hence, validation of *de novo* driver mutations using appropriate *in vivo* model systems is important. The EPH receptor A3 (*EPHA3*) tyrosine kinase is among the most frequently mutated cancer genes in human lung adenocarcinomas. However, we still lack mouse genetic studies to unequivocally validate its previously assigned putative tumor suppressor function in human lung adenocarcinomas.

**Results**

Here, the authors test the applicability of *EphA3*-null mice to address the functional importance of EPHA3 in mutant Kras- or p53-loss-driven mouse lung adenocarcinomas. The study shows that constitutive loss of *EphA3* does not alter mutant Kras-driven lung adenocarcinoma progression, nor the histopathology or latency of p53-loss-driven adenocarcinomas. Moreover, the study identifies EPHA3 as a receptor that is expressed in embryonic lung mesenchyme and describes subtle lung morphogenesis gene expression changes in *EphA3* heterozygous embryonic lungs. No gross phenotypic changes in morphogenesis-related functions are detected in *EphA3* heterozygous or null embryonic lungs.

**Implications and future directions**

This study highlights the importance of creating appropriate model systems to study the *in vivo* functional relevance of putative cancer drivers, such as EPHA3. Our studies utilizing *Epha3*-null mice fail to validate a putative tumor suppressor function for *EPHA3* in human lung cancer. Furthermore, the overlapping expression pattern of EPH receptors detected in the developing mouse embryonic lung might imply functional redundancy. Therefore this study provides an incentive to the design of sophisticated, possibly tissue-specific, knock-in or conditional mouse models using genome-editing tools such as the prokaryotic type II CRISPR/Cas-system to elucidate the role of EPHA3 mutations during lung tumorigenesis *in vivo*.

Genetically engineered mouse models (GEMMs) are the most widely applied and functionally validated *in vivo* models of human lung cancer, in particular to validate gene cooperation concomitant with conditional expression of the oncogenic *Kras* gene (Jackson et al., 2001; Jackson et al., 2005; Ji et al., 2007; Schramek et al., 2011; Snyder et al., 2013). Importantly, murine clinical studies have shown that oncogenic signaling in *Kras*-driven GEMMs is crucially defined by the cooperating tumor suppressor, with loss of liver kinase B1 (*Lkb1*) conferring different therapeutic responses compared with loss of *Trp53* (Chen et al., 2012). Despite convincing data suggesting a tumor suppressor role for EPHA3 during lung tumor progression, thus far no studies have addressed its *in vivo* functional role. We therefore decided to utilize the *EphA3*-null mice to test the effect of constitutive loss of *EphA3* on lung ADC progression driven by mutant *Kras* (LSL-*Kras^G12D^*^/+^) (Jackson et al., 2001) and loss of *Trp53* (*p53^fl/fl^*) (Marino et al., 2000), hereafter referred to as *Kras* and *p53*. Our data shows that the constitutive loss of *EphA3* does not alter the progression of murine ADC in either of these models. Moreover, despite clear evidence for *EphA3* expression in the developing lung, similar to key regulators of morphogenesis known to regulate lung tumorigenesis (Clark et al., 2001; Snyder et al., 2013; Yin et al., 2013), an analysis of selected EphA family receptors shows that *EphA3* has a non-unique or minimal function during lung morphogenesis. Our study thus provides an incentive for rational design of novel GEMMs to unequivocally assign the role of *EPHA3* during lung tumorigenesis *in vivo*.

## RESULTS

### Constitutive loss of *EphA3* does not accelerate mutant *Kras*- or *p53*-loss-driven lung tumorigenesis

**Fig. 1.**
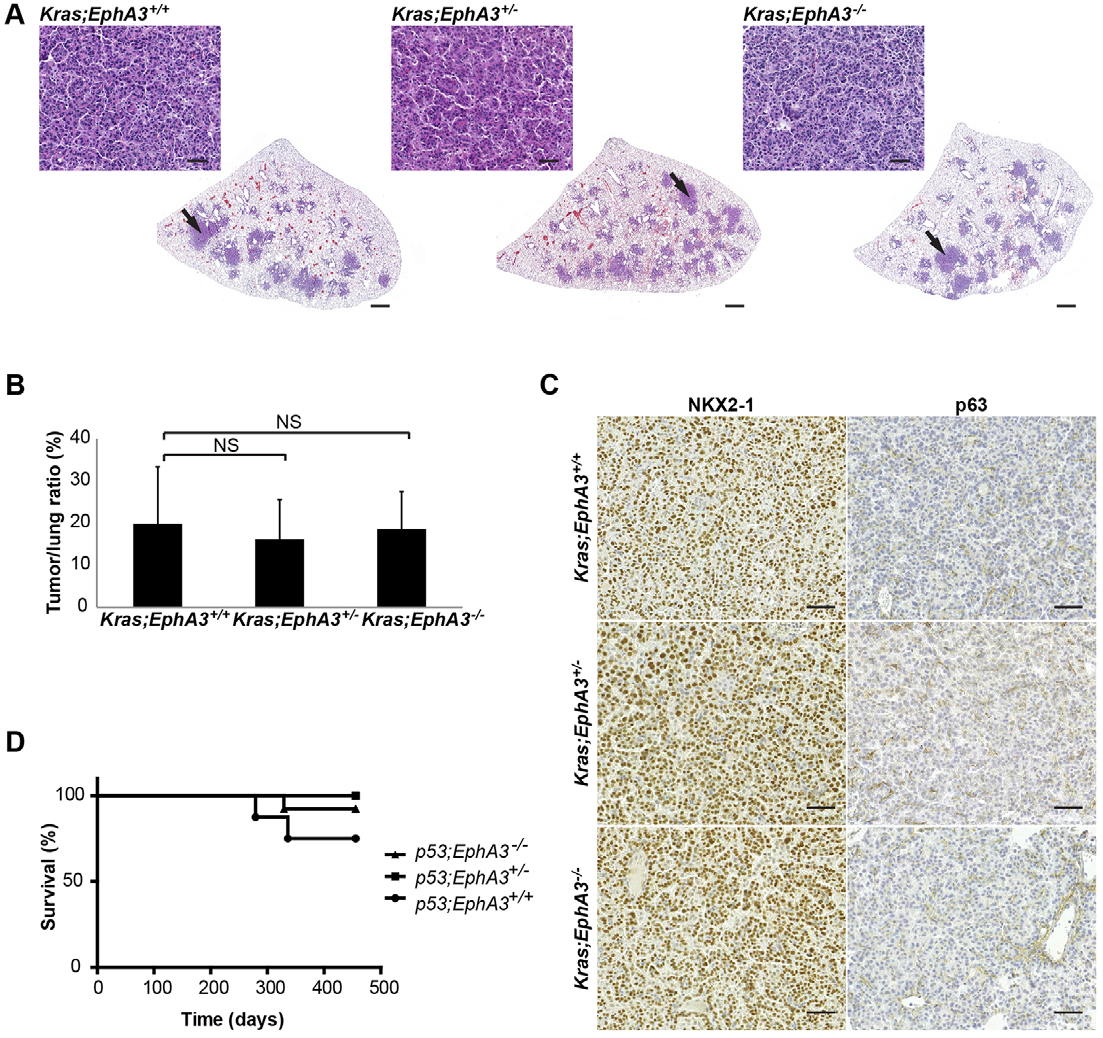
**Constitutive loss of *EphA3* does not alter mutant *Kras*-driven or *p53* loss-induced lung ADCs.** (A) Representative sections (stained with H&E) depicting the tumor burden in *Kras;EphA3*^+/+^, *Kras;EphA3*^+/−^ and *Kras;EphA3*^−/−^ lungs 19 weeks post CMV-AdCre infection (3.3×10^7^ PFUs) show no difference between the genotypes. Black arrows indicate the site of magnified images. (B) Average tumor-to-lung area at 19 weeks after CMV-AdCre infection of the *Kras;EphA3*^+/+^, *Kras;EphA3*^+/−^ and *Kras;EphA3*^−/−^ lungs. Two separate lung regions were used for tumor burden analysis. *n*=9 for *Kras;EphA3*^+/+^, *n*=16 for *Kras;EphA3*^+/−^ and *n*=8 for *Kras;EphA3*^−/−^. Results are mean±s.d. (C) Immunohistochemical analysis of the ADC marker NKX2-1 and squamous cell carcinoma marker p63 in *Kras;EphA3*^+/+^, *Kras;EphA3*^+/−^ and *Kras;EphA3*^−/−^ tumors, indicating positive nuclear staining for NKX2-1 and negative for p63. (D) Survival curves of *n*=7 for *p53;EphA3*^+/+^, *n*=11 for *p53;EphA3*^+/−^ and *n*=13 for *p53;EphA3*^−/−^ mice treated with CMV-AdCre (3.3×10^8^ pfu). Mice were monitored for 15 months, during which one out of seven *p53;EphA3*^+/+^ and two out of 13 *p53;EphA3*^−/−^ mice died due to CMV-AdCre-induced ADC formation. NS, *P*>0.05 (Student’s *t*-test). Scale bars: 1 mm for whole lung lobes; 50 μm for magnified images.

To test the hypothesis that EPHA3 acts as a lung tumor suppressor, we used a previously described constitutive *EphA3*-null mouse model (Stephen et al., 2007; Vaidya et al., 2003). *EphA3*-null mice did not show any marks of reduced survival during a 1-year followup period, indicating that mere *EphA3* loss does not drive tumorigenesis. We therefore assessed whether *EphA3* loss could accelerate tumorigenesis induced by conditional alleles known to initiate lung ADC, following a classic multi-allele paradigm. These ‘first hit’ conditional models comprised mutant *Kras* (Jackson et al., 2001) and loss of *p53* (Marino et al., 2000), which are also common drivers of human disease found in at 17% and 35% of ADCs, respectively (COSMIC, 2014; http://cancer.sanger.ac.uk/cancergenome/projects/cosmic/). In lung ADC, *EPHA3* mutations show a statistically significant tendency towards co-occurrence with mutations in *TP53* (*P*<0.01) and occasional, but not statistically significant, co-occurrence with *KRAS* mutations (supplementary material Fig. S1A). We established cohorts of 8–16 mice for each genetic combination (homozygous *p53* or heterozygous *Kras* with wild-type *EphA3*, or homozygous or heterozygous null *EphA3*). Lung-specific deletion of conditional alleles was achieved through intranasal inhalation of adenoviral Cre recombinase (CMV-AdCre), affording transduction of bronchiolar and alveolar progenitor cells, to initiate carcinoma progression. The infection efficacy was confirmed with a dual fluorescence *mT/mG* Cre-reporter strain that monitors *in vivo* integration efficiency through activating a Cre-dependent switch from membrane-tagged Tomato to GFP (supplementary material Fig. S1B) (Muzumdar et al., 2007). Tumor burden analysis at 19 weeks after CMV-AdCre infection showed that constitutive loss of *EphA3* did not alter mutant *Kras*-driven lung ADC progression (Fig. 1A,B; supplementary material Fig. S1C). Similar to previous findings in murine *Kras* lung cancer studies (Jackson et al., 2001), all *EphA3* genotype cohorts displayed distinct types of progressive lesions, including epithelial hyperplasia, adenomas and ADCs. In addition, we detected previously described profound inflammatory responses as infiltrations of macrophages and neutrophils (supplementary material Fig. S1C) (Ji et al., 2006). Furthermore, analysis of histopathology and NKX2-1 and tumor protein 63 (p63) biomarker expression to respectively depict ADC and SCC tissue, showed that constitutive absence of *EphA3* did not alter the tumor histology (Fig. 1C). We further found that the loss of *EphA3* did not alter the latency of *p53*-loss-driven ADCs (Fig. 1D). Thus, the constitutive absence of EPHA3 expression does not accelerate mutant *Kras*- or *p53*-loss-driven lung tumorigenesis.

### Mesenchymal expression of *EphA3* suggests that it has a functional role during lung development

**Fig. 2.**
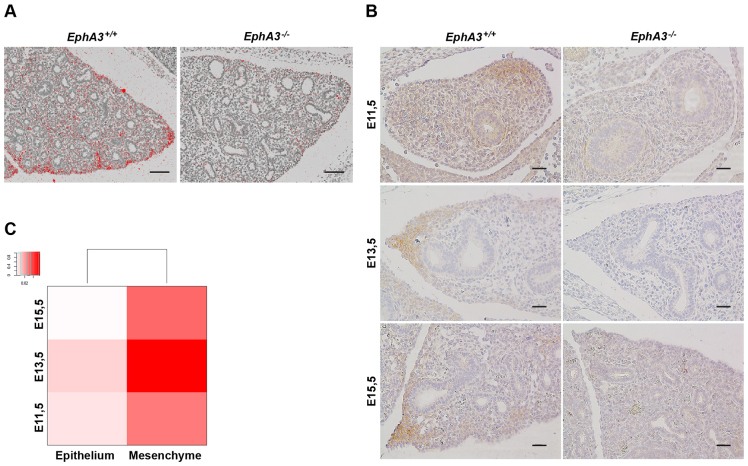
**Mesenchymal expression of *EphA3* during lung development.** (A) *In situ* hybridization analysis of *EphA3* expression in embryonic mouse lungs at E14.5, showing its distal mesenchymal expression. (B) Immunohistochemical analysis of EPHA3 protein in embryonic mouse lungs at E11.5, E13.5 and E15.5 confirms the distal mesenchymal expression. (C) Quantitative PCR analysis of epithelial and mesenchymal mRNA expression of *EphA3* at E11.5, E13.5 and E15.5 indicating strongest expression at E13.5. The inset shows the distribution of the samples within the observed expression values. Scale bars: 100 μm.

As previous studies have indicated a role for *EphA3* in embryonic development, we undertook a detailed expression analysis of *EphA3* in the developing mouse lung. Both RNA *in situ* hybridization (Fig. 2A) and immunohistochemical staining (Fig. 2B) demonstrated expression of *EphA3* in the distal mesenchyme of the embryonic lung. The specificity of the EPHA3 antibody was confirmed by absence of detectable immunohistochemical staining in *EphA3*-null embryos (Fig. 2A,B), as well as a decreased signal in hTERT-RPE1 cells treated with *EPHA3* small interfering RNA (siRNA) (supplementary material Fig. S1D). Expression of EPHA3 in the developing lung was detected during embryonic ages E11.5 to E15.5 (Fig. 2B,C), which falls into the pseudoglandular (E9.5–E16.5) stage of murine lung development. During this stage, the newly generated primary lung buds develop into a complex branched tree-like structure ending in thousands of epithelial terminal tubules, accompanied by continued mesenchymal growth around the growing epithelia (Morrisey and Hogan, 2010). Based on this mesenchymal expression in the developing mouse lungs we hypothesized that EPHA3 might function during the pseudoglandular stage of lung development.

### Expression of multiple EPH receptors in the developing mouse lung suggests involvement in lung morphogenesis

**Fig. 3.**
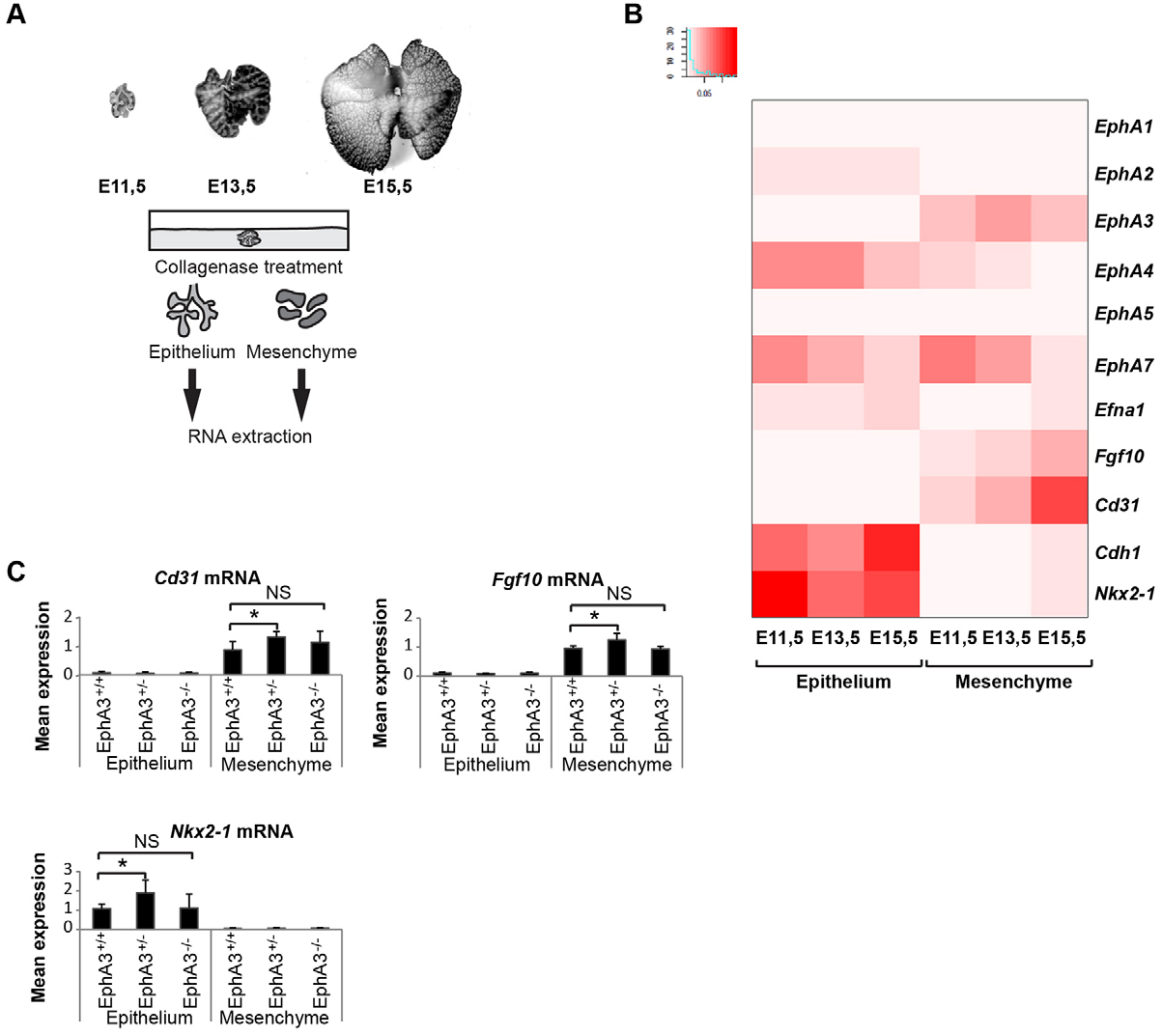
**Embryonic lung gene expression analysis of selected EphA family and morphogenesis genes.** (A) Schematic workflow describing the mRNA expression analysis of E11.5, E13.5 and E15.5 embryonic lung epithelium and mesenchyme. (B) Average mRNA expression levels of selected EphA family receptors and *Efna1* ligand. Expression of epithelial *Nkx2-1* and *Cdh1*, mesenchymal *Fgf10* and endothelial *Cd31*, correlates with their known expression patterns. E11.5, *n*=2, E13.5 and E15.5, *n*=4. The inset shows the distribution of the samples within the observed expression values. (C) Comparative mRNA expression analysis of *EphA3*^+/+^, *EphA3*^+/−^ and *EphA3*^−/−^ dissected embryos at E13.5 shows a minor but statistically significant difference between *EphA3*^+/+^and *EphA3*^+/−^ in epithelial *Nkx2-1* and mesenchymal *Fgf10* and *Cd31* expression. Results are mean±s.d. (*n*=4). *P<0.05 (Student’s *t*-test).

We next asked how mRNA expression of *EphA3* during murine lung morphogenesis might correlate with or impact on mRNA expression of other EphA receptors in epithelial and mesenchymal cells at E11.5, E13.5 and E15.5 of lung development (Fig. 3A). At E11.5 we performed quantitative PCR (q-PCR) expression analysis on both proximal and distal epithelium and mesenchyme. At E13.5 and E15.5, the analysis was restricted to the distal regions, approximating the terminal epithelial buds and their surrounding mesenchyme. We found that among the studied EphA receptors, only expression of *EphA3* was restricted to the developing lung mesenchyme, and closely overlapped with expression of known mesenchymal *Fgf10* and endothelial *Cd31* (also known as *Pecam1*) genes (Fig. 3B; supplementary material Fig. S2A). *EphA7* expression was detected both in the mesenchyme and epithelia, and was the only other EphA receptor co-expressed with *EphA3* in the mesenchyme (Fig. 3B). Importantly, we did not detect any compensatory changes in *EphA7* expression levels in the heterozygous or homozygous *EphA3*-null embryonic lungs (supplementary material Fig. S2B). *EphA2* and *EphA4* were found to be expressed mainly in epithelial cells, whereas expression of *EphA1* and *EphA5* was absent in both tissue compartments (Fig. 3B). These results correlate with *in situ* hybridization data described by the Allen Institute for Brain Science, with the exception of *EphA1*, for which moderate expression was detected in murine lung epithelia (Allen Institute for Brain Science, 2013). We further confirmed the epithelial expression of the known ligand of EPHA3, ephrin-A1 (encoded by *Efna1*), at E11.5, E13.3 and E15.5 (Fig. 3B; supplementary material Fig. S2C). Finally, postnatal murine lung expression analysis revealed very low *EphA3* and *EphA7* expression levels when compared to that of the embryonic mesenchyme (E13.5), whereas *EphA2* and *EphA4* expression were higher in the adult tissue (supplementary material Fig. S3A). Taken together, our data identifies *EphA3* as a mesenchymal EPH receptor and suggests that its ligand ephrin-A1 is expressed in the adjacent branching epithelia. Furthermore, the low expression of *EphA3* in adult tissue suggests that EPHA3 is absent or has a minimal role in postnatal lung homeostasis.

### *EphA3* heterozygosity is associated with altered expression of branching morphogenesis and vasculogenesis genes

Next, we investigated whether constitutive loss of *EphA3* affected the mRNA expression of known lung morphogenesis genes. A targeted q-PCR analysis of known regulators of lung morphogenesis identified a small but significant increase in the expression of *Nkx2-1* in heterozygous *EphA3* embryonic epithelium at E13.5 when compared with wild-type tissue (Fig. 3C). Furthermore, similar expression increases were detected in endothelial *Cd31* and mesenchymal *Fgf10* (Fig. 3C). In contrast, analysis at E15.5 failed to show statistically significant expression differences for these three genes (supplementary material Fig. S3B) suggesting that any role for *EphA3* during pseudoglandular lung development is transitory. Taken together, the partial loss of *EphA3* appears to induce subtle and transitory alterations in epithelial *Nkx2-1*, endothelial *Cd31* and mesenchymal *Fgf10* mRNA expression, suggesting that EPHA3 function might modulate lung morphogenesis.

### Constitutive loss of *EphA3* does not overtly affect murine lung morphogenesis

**Fig. 4.**
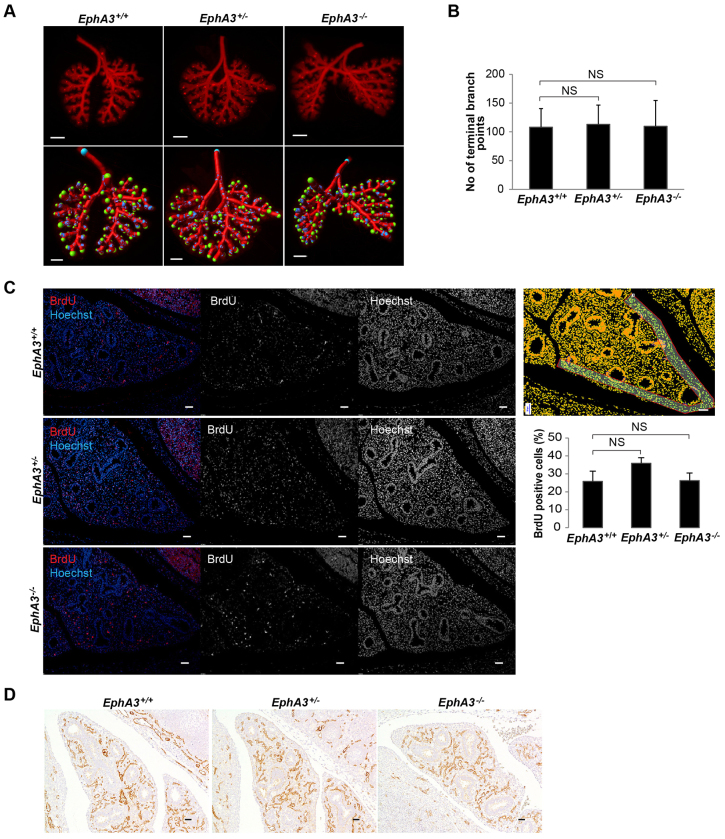
**Constitutive loss of *EphA3* does not alter morphogenesis of murine lungs.** (A) Representative E-cadherin whole-mount images of *EphA3*^+/+^, *EphA3*^+/−^ and *EphA3*^−/−^ embryonic lungs at E13.5 and corresponding images from branch end-point analyses, indicating end points in green. (B) Mean±s.d. of the branch end-point number from *EphA3*^+/+^, *EphA3*^+/−^ and *EphA3*^−/−^ lungs at E13.5 shows no difference between *EphA3*^+/−^ and *EphA3*^−/−^ embryonic lungs when compared to *EphA3*^+/+^ lungs; *n*=6 in all three genotypes. (C) Representative images from BrdU and nuclear Hoechst immunohistochemical staining in *EphA3*^+/+^, *EphA3*^+/−^ and *EphA3*^−/−^ embryonic lungs at E13.5. Segmentation of representative images was performed to calculate distal-mesenchyme-specific BrdU proliferation analysis. Mean±s.d. values of the amount of BrdU-positive cells relative to the total number of Hoechst-stained nuclei shows no difference between *EphA3*^+/+^, *EphA3*^+/−^ and *EphA3*^−/−^ embryonic lungs at E13.5. *n*=3 for *EphA3*^+/+^ and *EphA3*^−/−^; *n*=1 for *EphA3*^+/−^. Analysis was done on two separate regions of the embryonic lungs. NS, *P*<0.05 (Student’s *t*-test). (D) Representative images of CD31-positive endothelial cells of *EphA3*^+/+^, *EphA3*^+/−^ and *EphA3*^−/−^ embryonic lungs at E13.5. Scale bars: 300 μm in E-cadherin whole-mount images; 200 μm in the branch end-point images; and 100 μm in BrdU images.

We next asked whether the constitutive loss of *EphA3* was directly associated with altered lung branching morphogenesis. We first performed a quantitative analysis of lung branch end-points at E13.5 by E-cadherin whole-mount immunohistochemistry staining and optical projection tomography (OPT) to visualize branching epithelia. The number of terminal branches was found to be identical in *EphA3* heterozygous (average 113) and null embryonic lungs (average 110) when compared to age-matched littermate controls (average 108) (Fig. 4A,B). Additional qualitative analysis using E-cadherin-stained E11.5 and E15.5 whole-mount lungs further confirmed that EPHA3 does not overtly affect lung branching morphogenesis (supplementary material Fig. S3C). Next, we assessed whether loss of *EphA3* was associated with an alteration in distal mesenchymal cell proliferation. Analysis of *in vivo* BrdU incorporation showed that there was no statistically significant increase in the percentage of mesenchymal S phase cells in *EphA3* heterozygous (36%) or *EphA3*-null (26%) lungs at E13.5 when compared to littermate controls (26%) (Fig. 4C). Finally, we studied whether the pulmonary vasculature formation was altered by loss of *EphA3* by analyzing CD31 expression at E13.5. In both *EphA3*-null and heterozygous lungs, the number of CD31-positive endothelial cells at E13.5 was identical to that of the controls (Fig. 4D). Taken together, the data presented here show that constitutive loss of *EphA3* does not overtly alter murine lung morphogenesis.

## DISCUSSION

The functional validation of *de novo* mutations identified in lung cancer sequencing efforts is a prerequisite for the development of novel targeted therapies. *EPHA3* is among the most frequently mutated RTKs in human lung ADCs, and has been assigned a candidate tumor suppressor role based on its mutation spectrum and findings from *in vitro* and *in vivo* studies (Lahtela et al., 2013; Lisabeth et al., 2012; Vail et al., 2014; Zhuang et al., 2012). However, the actual role of EPHA3 during lung tumor progression has not been investigated nor validated using GEMMs. Our previous findings linked loss of EPHA3 to p53 activation (Lahtela et al., 2013), and *EPHA3* and *TP53* point mutations display statistically significant co-occurrence in lung ADC (supplementary material Fig. S1A). We hence asked whether the absence of *EphA3* enhanced the incidence of *p53*-loss-driven lung cancer progression. Additionally, we asked whether loss of *EphA3* accelerated lung ADC progression caused by the commonly mutated *Kras* oncogene. We here show that the constitutive absence of EPHA3 does not affect tumor progression and histopathology of both *p53*-loss- and mutant *Kras*-driven lung ADCs. Thus, *EphA3*-null mice fail to validate a putative tumor suppressor function for *EPHA3* in human lung cancer, perhaps owing to functional redundancy between murine EphA receptors expressed in adult lungs. Interestingly, sequencing of murine small cell lung cancer (SCLC) tumors initiated by loss of *p53* and retinoblastoma 1 (Rb1) revealed that there were recurrent somatically acquired *EphA5* and *EphA7* mutations (McFadden et al., 2014). This means that there is a strong case for further lung tumorigenesis studies to study the role of EphA receptor biology in GEMMs, and in particular the physiological role of *EphA5* and *EphA7*.

Re-activation of EPH-receptor–ephrin pathways, generally known to contribute to cell sorting and tissue patterning in embryonic development, has been causally linked with tumorigenesis (Nievergall et al., 2012). Moreover, expression of key regulators of embryonic lung morphogenesis, *Fgf9* and *Fgf10* (Colvin et al., 2001; Min et al., 1998; White et al., 2006), has been shown to trigger ADC and adenoma progression, respectively (Clark et al., 2001; Yin et al., 2013). Thus far, of all EphA receptors and ligands, only a role of the ephrin-B2 ligand has been described during lung development. Specifically, ephrin-B2 has been shown to regulate alveolar epithelial and endothelial viability and vascular growth in hyperoxic rats (Vadivel et al., 2012), as well as pulmonary compliance in mice (Bennett et al., 2013). Our current data shows that *EphA3* is expressed specifically in the mesenchymal distal lung tips during the pseudoglandular stage of branching morphogenesis, albeit at low levels. However, whereas for example the removal of *Fgf10* results in dramatic defects in lung organogenesis (Min et al., 1998), partial loss of *EphA3* appears to induce only subtle increases in epithelial *Nkx2-1*, endothelial *Cd31* and mesenchymal *Fgf10* mRNA expression levels. Furthermore, no macroscopic phenotypic effect on lung epithelial branching, mesenchymal cell proliferation, or abundance and localization of CD31-positive endothelia was measured. This lack of a discernible phenotype might indicate: (1) lack of an overt, or a different, role for EPHA3 in the murine lung; (2) functional redundancy between lung-expressed EphA receptors; or (3) a partial penetrance of the *EphA3*-null genotype.

Of the selected EphA receptors, we found that only *EphA7* was co-expressed with *EphA3* in the lung mesenchyme. Interestingly, a recent study has suggested that there is functional compensation of *EphA3* loss by *EphA7* co-expression during palate development, as compound homozygous mutation of *EphA3* and *EphA4* failed to cause defective midfacial development (Agrawal et al., 2014). Furthermore, a truncated form of EPHA7 has been reported to act as a tumor suppressor in follicular lymphoma (Oricchio et al., 2011), and it would thus be interesting to study its potential role in lung tumor suppression in conjunction with *EphA3* loss of function.

Taken together, we report that loss of *EphA3* does not lead to measurable effects on lung ADC progression, nor lung morphogenesis, in the applied constitutive null GEMM. Importantly, we cannot exclude the possibility that other EphA receptors co-expressed in the (developing) lung, most notably *EphA7*, can compensate for the decreased expression of *EphA3*. Our findings therefore provide an incentive to perform a rational design of tissue-specific knock-in or conditional mouse models to unequivocally assign the role of *EPHA3* mutation or loss of expression, possibly in the context of compound EPHA-ephrin network mutations, on lung tumorigenesis *in vivo*. In this respect, the ability to apply prokaryotic type II CRISPR/Cas genome editing tools to introduce somatic germline mutations in mice (Sanchez-Rivera et al., 2014; Yang et al., 2013) provides promise for future tumor modeling approaches.

## MATERIALS AND METHODS

### Mouse cohorts and tissue preparation

Animal studies were carried out in accordance with guidelines from the Finnish National Board of Animal Experimentation, and were approved by the Experimental Animal Committee of the University of Helsinki and the State Provincial Office of Southern Finland (License number ESAVI-2010-04855/Ym-23). *EphA3*-null mice lacking a genetic region encompassing the first exon of *EphA3* were previously described (Stephen et al., 2007; Vaidya et al., 2003). Mice carrying a conditional mutant allele of *Kras* (LSL-*Kras*^G12D/+^) (Jackson et al., 2001) or a loss-of-function allele of *Trp53* (*p53*^fl/fl^) (Marino et al., 2000) were purchased from The Jackson Laboratory. *EphA3*-null mice were bred with *Kras* and *p53* mice to generate the study cohorts, and were maintained on a mixed genetic background using littermates as controls. Multiple litters of the same age were used to provide sufficient numbers of each genotype. Lung tumorigenesis was initiated by infecting mice at 6–10 weeks of age with 3.3×10^7^ (*Kras*) or with 3.3×10^8^ (*p53*) plaque-forming units (PFUs) of recombinant adenovirus expressing the Cre recombinase (University of Turku, Finland), using intranasal instillation as described elsewhere (Jackson et al., 2001). Viruses were administered in a Biosafety Level 2+ room according to the guidelines of the Finnish Board for Gene Technology. Lungs from mice were fixed in 4% formaldehyde, and all lobes were embedded in paraffin.

### Tumor burden analysis and survival curves

Lungs from *Kras;EphA3*^−/−^, *Kras;EphA3*^+/−^ and *Kras;EphA3*^+/+^ mice at 19 weeks post infection were processed as described above and paraffin sections (4 μm) were cut from two distinct zones (middle and bottom) of each paraffin block, thus generating two sections each representing the whole lung surface area, which were stained with H&E. Whole slide scans of the H&E-stained lung sections were acquired with a Pannoramic 250 3DHISTECH (3DHISTECH Kft., Budapest, Hungary) digital slide scanner with a 20× objective. Whole slide images were assessed for tumor burden using the Tissue studio image analysis solution of the Definiens Developer XD 64 2.1 software (Definiens, Munich, Germany). The histopathology of the lesions and inflammatory infiltrations were diagnosed by an expert pathologist. Long-term follow up of infected *p53;EphA3*^−/−^, *p53;EphA3*^+/−^ and *p53;EphA3*^+/+^ mice was performed until 15 months, and mice were killed when showing labored breathing. Sections (4 μm) were cut from two distinct zones (middle and bottom) of each paraffin block, stained with H&E and qualitatively analyzed for tumor appearance. Kaplan-Meier survival curves were generated using Prism (GraphPad Software, Inc., San Diego, CA).

### Histology and immunohistochemistry

Immunohistochemistry was performed on paraffin-embedded sections (4 μm). Sections were dehydrated and antigenic epitopes were exposed by heating in 10-mM citrate buffer (pH 6.0) or by incubation in 0.05% trypsin at +37°C. Sections were incubated with the following antibodies: anti-NKX2-1 (Abcam, Cambridge, UK); anti-p63 (Abcam, Cambridge, UK); anti-EPHA3 (Invitrogen/Thermo Fisher Scientific Inc., Waltham, MA); anti-CD31 (Becton, Dickinson and Company, Franklin Lakes, NJ); anti-GFP (polyclonal rabbit serum 8 mg/ml, generated in house). Primary antibody staining was detected using Bright vision poly-horseradish peroxidase (HRP)-conjugated goat anti-rabbit IgG (ImmunoLogic, Duiven, The Netherlands), HRP-conjugated goat anti-rat IgG (Invitrogen/Thermo Fisher Scientific Inc., Waltham, MA) and 3,3′-diaminobenzidine (DAB) (Immunologic, Duiven, The Netherlands) or Alexa-Fluor-488-conjugated anti-rabbit IgG (Life Technologies/Thermo Fisher Scientific Inc., Waltham, MA). Sections were counterstained with Mayers hemalum solution (Millipore, Billerica, MA) or Hoechst 33342 dye (Invitrogen/Thermo Fisher Scientific Inc., Waltham, MA). Image acquisition was performed either using a Nikon 90i Eclipse microscope (Nikon Instruments Europe BV, The Netherlands) and DS-Fi2 5 MP camera, or a Pannoramic 250 3DHISTECH (3DHISTECH Kft., Budapest, Hungary) digital slide scanner with a 20× objective.

### *In situ* hybridization

Radioactive *in situ* hybridization was performed on paraffin sections according to the standard protocols using probes labeled with ^35^[S]-UTP. Dark-field images were inverted, linearly thresholded and combined with brightfield images in Adobe Photoshop CS6 (Adobe Systems Software, Dublin, Ireland). The mouse *EphA3* probe was an 817-bp fragment (nucleotides 658–1474) inserted into pGEM-3Zf- vector. The mouse *Efna1* probe was a 402-bp fragment (nucleotides 20–421) inserted into pGEM-3Zf-vector. The *Fgf10* probe was a 584-bp fragment (nucleotides 11–579) inserted into Bluescript KSII+ vector.

### BrdU proliferation assay

A timed pregnant mouse was injected with 5-bromo-2′-deoxyuridine (BrdU) (Sigma, St Louis, MO) and killed 4 hours later to harvest embryos at embryonic age of 13.5. Embryos were fixed in 4% formaldehyde and embedded in paraffin. Sections (4 μm) were cut from two distinct zones of the embryonic lungs. BrdU-positive cells were detected using anti-BrdU antibody (Cell Signaling Technology, Danvers, MA) and counterstained with Hoechst 33342 using standard immunohistochemical methods. Image acquisition was performed with a Nikon 90i Eclipse microscope (Nikon Instruments Europe BV, The Netherlands) and DS-Fi2 5 MP camera. Image analysis was done using NIS-Elements AR 4.2 software (Nikon Instruments Europe BV, The Netherlands).

### Preparation of embryonic lung tissue

Embryonic lung dissection and epithelial and mesenchymal cell separation was performed as previously described (del Moral and Warburton, 2010), with small modifications. Briefly, pregnant mice were killed to harvest embryos at E11.5, E13.5 and E15.5 by CO_2_ administration. Collected embryos were dissected under a stereoscopic microscope in a glass Petri dish immersed in PBS. Isolated lungs were then transferred to 24-well plates containing CO_2_-independent medium (Gibco by Life Technologies/Thermo Fisher Scientific Inc., Waltham, MA). Epithelial and mesenchymal tissues were separated by treating with 10 mg/ml collagenase (collagenase from *Clostridium histolyticum*, Sigma) in CO_2_-independent medium at 37°C for 20 minutes. Enzymatic degradation was stopped by adding CO_2_-independent medium supplemented with 5 U/ml RNase-free DNase (RQ1 RNase free DNase, M6101, Promega, WI). Depending on the embryonic age, mesenchymal and epithelial cells from one to five embryos were used to reach high enough RNA yields.

### Quantitative PCR analysis

Normal adult lung tissue was homogenized using a Precellys homogenization kit (Bertin Technologies, Montigny-le-Bretonneux, France). Total RNA was extracted using NucleoSpin RNA II kit (MACHEREY-NAGEL, Düren, Germany) and quantified using NanoDrop 1000 (Thermo Fisher Scientific Inc.). Complementary DNA (cDNA) was synthesized from the extracted RNA using a High-capacity cDNA reverse transcription kit (Applied Biosystems by Life Technologies/Thermo Fisher Scientific Inc.). The q-PCR amplification was performed using iQ™ SYBR^®^ Green Supermix (Bio-Rad, Hercules, CA) or iQ™ Supermix (Bio-Rad) and CFX384 Touch™ Real-Time PCR Detection System C1000 Touch (Bio-Rad). The following TaqMan^®^ probes were used to measure the *EPHA3* expression in hTERT-RPE1 cells: EPHA3 Hs00739096_m1 and RPL19 Hs02338565_gH (Applied Biosystems by Life Technologies/Thermo Fisher Scientific Inc.). q-PCR primers were designed to flank exon-exon boundaries and to give specific amplification. Following 3 minutes denaturation at 95°C, 40 cycles of 15 seconds at 95°C and 1 minute in 60°C were run. A melting curve ranging from 57°C to 95°C was included in every analysis to confirm the specific amplification. Primer details are listed in supplementary material Table S1. An exponential expression (ΔCq Expression) was obtained with formula ΔCq Expression=2^−ΔCq^, where ΔCq=Cq (target) − Cq (reference). The average of the ΔCq expression values of the specific genotypes and time points were visualized with heatmaps generated using an R statistical programing language heatmap function from the Heatplus Bioconductor package. We used R version 2.15.3 freely available at http://www.r-project.org/.

### Whole-mount immunohistochemistry and optical tomography scanning

Sample processing and whole-mount immunohistochemistry of dissected embryonic lungs at E11.5–E15.5 were performed as described previously (Alanentalo et al., 2007). Briefly, fixed lungs were dehydrated with methanol followed by rehydration and processing to immunohistochemical staining. Localization of anti-E-cadherin (Cell Signaling Technology) was detected either by fluorescently labeled secondary antibody conjugated to Alexa-Fluor-594-conjugated anti-rabbit IgG (Life Technologies/Thermo Fisher Scientific Inc.) or visualized by using the chromogenic DAB substrate (Immunologic, Duiven, The Netherlands) following the incubation with poly-HRP-conjugated anti-rabbit IgG antibody (Immunologic, Duiven, The Netherlands). Fluorescently labeled lungs were processed for OPT scanning as described previously (Alanentalo et al., 2007), using a Bioptonics OPT 3001M Scanner. Three-dimensional (3D) visualization and branch end-point analysis was performed with Imaris 3D and 4D data software, using the Filament analysis function (Bitplane AG, Switzerland). Chromogenically stained samples were imaged using a Leica MZFLIII stereomicroscope (Leica, Germany) and Colorview camera (Software imaging system, Olympus, Japan).

### Cell culture and transfections

hTERT-RPE1 cells (Clontech, CA) were maintained in DMEM with F-12 (Sigma) containing 10% FBS, 2 mM L-glutamine, 0.348% sodium bicarbonate and penicillin-streptomycin (all Gibco by Life Technologies/Thermo Fisher Scientific Inc.), following the manufacturer’s recommendations. For gene knockdown, hTERT-RPE1 cells were treated with 50 nM pooled siRNAs against *EPHA3* (GE Dharmacon, Denver, CO) or siCONTROL non-targeting siRNA #3 (SiCtrl; GE Dharmacon, Denver, CO) after transfection with Oligofectamine reagent (Invitrogen/Thermo Fisher Scientific Inc.) on 15-cm culture dishes. One fifth of the transfected cells was pelleted for RNA extraction and *EPHA3* mRNA quantification.

## Supplementary Material

Supplementary Material
