# Supplementary material for: The putative tumor suppressor gene EphA3 fails to demonstrate a crucial role in murine lung tumorigenesis or morphogenesis
Source: Dis Model Mech. 2015 Feb 20;8(4):393–401. doi: 10.1242/dmm.019257 (PMC4381338; doi:10.1242/dmm.019257)
Supplement: Supplementary Material [file supp_8_4_393__index.html]

The putative tumor suppressor gene EphA3 fails to demonstrate a crucial role in murine lung tumorigenesis or morphogenesis — Supplementary Material 

# The putative tumor suppressor gene *EphA3* fails to demonstrate a crucial role in murine lung tumorigenesis or morphogenesis

## DMM019257 Supplementary Material

**Files in this Data Supplement:**

- **Supplementary Material**
