# Supplementary material for: The putative tumor suppressor gene EphA3 fails to demonstrate a crucial role in murine lung tumorigenesis or morphogenesis
Source: Dis Model Mech. 2015 Feb 20;8(4):393–401. doi: 10.1242/dmm.019257 (PMC4381338; doi:10.1242/dmm.019257)

SUPPLEMENTARY MATERIAL

Supplementary Table 1. List of primers used in the quantitative PCR analysis of mouse epithelial and mesenchymal cells. Primer sequences for *EphA1* and *EphA4* were from the indicated references.

Gene	Primer sequence	Primer efficiency
EphA1 (Forward)	CAAGATTGCAAGACTGTGGC (Abdul-Aziz et al., 2009)	E=98.3% R^2=0.949 Slope= -3.363
EphA1 (Reverse)	CCTCCCACATTACAATCCCA (Abdul-Aziz et al., 2009)	
EphA2 (Forward)	GTCTATAAAGGGACGCTGAAGG	E=96.3% R^2=0.987 Slope= -3.414
EphA2 (Reverse)	CGCTCCATTCTCCATGTACTC	
EphA3 (Forward)	GAGACAGTATGCCGCAGTCA	E=99.0% R^2=1.000 Slope= -3.346
EphA3 (Reverse)	GCCTCTTGCTCTCAAAATGG	
EphA4 (Forward)	CCGAAGCAGCCTACACTACC (Andersson et al., 2011)	E= 98.7 R^2= 0.996 Slope= -3.354
EphA4 (Reverse)	GCCAGCAGTCCAGCATTAAC (Andersson et al., 2011)	
EphA5 (Forward)	AGTGACAGTGGGAGTCATCT	E=90.7% R^2=0.943 Slope= -3.566
EphA5 (Reverse)	GCAGTTTAATGTGCCCGTTATG	
EphA7 (Forward)	CAGCAGACGGGATTAGAGGA	E=100.4% R^2=0.988 Slope= -3.313
EphA7 (Reverse)	GATGACTCCATTGGGATGCT	
Efna1 (Forward)	CAGGAATCCCAGTGCTTGAA	E=94.2% R^2=0.998 Slope= -3.468
Efna1 (Reverse)	CAGCAGTGGTAGGAGCAATAC	
Efna5 (Forward)	GAGATGTTGACGCTGCTCTT	E=102.4% R^2=0.996 Slope= -3.266
Efna5 (Reverse)	TTCTGGGACAGAGTCCTCATAG	
Efnb2 (Forward)	CCAGACAAGAGCCATGAAGAT	E=74.6% R^2=0.975 Slope= -4.130
Efnb2 (Reverse)	TGCGATCCCTGCGAATAAG	
Fgf10 (Forward)	GCTGTTCTCCTTCACCAAGTA	E=95.1% R^2=0.969 Slope= -3.445
Fgf10 (Reverse)	ACTCCGATTTCCACTGATGTT	
Pecam1 (Forward)	GTGGTCATCGCCACCTTAATA	E=96.7% R^2=0.997 Slope= -3.404
Pecam1 (Reverse)	TTCTCGCTGTTGGAGTTCAG	
Nkx2.1(Forward)	CTACTGCAACGGCAACCTG	E=92.3% R^2=0.997 Slope= -3.522
Nkx2.1 (Reverse)	CCATGCCACTCATATTCATGC	
Cdh1 (Forward)	CAGGTCTCCTCATGGCTTTGC	E=89.6% R^2=0.994 Slope=-3.599
Cdh1 (Reverse)	CTTCCGAAAAGAAGGCTGTCC	
Rpl19 (Forward)	CGGGAATCCAAGAAGATTGA	E=93.4% R^2=0.997 Slope= -3.492
Rpl19 (Reverse)	TTCAGCTTGTGGATGTGCTC	

Supplementary figure legends

Supplementary Figure 1. (A) *EPHA3* mutations co-occur with mutations in common drivers of human lung adenocarcinoma. Grey bars represent patients with mutations in any of the three genes. Green mark indicates existence of point mutation in the assigned gene. Mutation information is based on the data from Cancer Genome Atlas Research Network (Cancer Genome Atlas Research Network, 2014) and was exported from cBioportal (Cerami et al., 2012;Gao et al., 2013). (B) Lungs from dual fluorescence mT/mG Cre-reporter mice at two weeks post CMV-AdCre infection (3,3 × 10^7 pfu) show Cre activity-induced GFP expression in bronchiolar [Br] and alveolar [Av] progenitor cells. Image acquisition was performed at 20x magnification. (C) Representative image of H&E stained lung section from *Kras;EphA3* lungs and the corresponding pseudo-colored image describing the tissue segmentation performed with Definiens software. The quantitation of cancer area [C] including epithelial hyperplasia, adenomas, and adenocarcinomas in all samples was measured in relation to the area of combined normal [N] and stromal regions [S] including infiltration of immune cells. Scale bars: 100 µm. (D) Validation of a polyclonal rabbit anti-EPHA3 antibody via siRNA-mediated downregulation of *EPHA3* in human hTERT-RPE1 cells. Cells were treated with *EPHA3* siRNA or siRNA control for 72 hours and processed for immunohistochemistry. Image acquisition was performed at 20x magnification. *EPHA3* mRNA quantitation confirms the knockdown upon siRNA treatment. Results are mean±s.d.

Supplementary Figure 2. (A) In situ hybridization analysis of *Fgf10* expression in embryonic mouse lungs at E14.5 shows mesenchymal expression. Scale bars: 100 µm. (B) Constitutive loss of *EphA3* does not trigger altered expression of *EphA7* in embryonic lung mesenchyme. Comparative expression analysis of *EphA3*$^{+/+}$, *EphA3*$^{+/-}$ and *EphA3*$^{-/-}$ embryonic lungs at E11.5, E13.5 and E15.5 shows statistically significant differences between *EphA3*$^{+/+}$and *EphA3*$^{-/-}$ only in epithelial *EphA7* expression. Results are mean±s.d. E11.5: n=2-3; E13.5 n=1-4; E15.5 n=3-4, *P < 0,05 (Student's t-test). (C) In situ hybridization of *Efna1* expression in embryonic mouse lungs at E14.5 shows epithelial expression. Scale bars: 100 µm.

Supplementary Figure 3. (A) *EphA3* is expressed at low levels in adult mouse lungs when compared with selected EphA receptors. Heatmap presentation of the comparative expression

analysis shows average expression values in *EphA3* -wild type and -null adult mouse lung and embryonic mesenchyme at E13.5 without scaling. Histogram presentation of the comparative expression analysis shows scaled average expression values in *EphA3* -wild type and -null adult mouse lung and embryonic mesenchyme at E13.5. Results are mean±s.d. N=3 for all four groups. (B) Comparative expression analysis of *EphA3*$^{+/+}$, *EphA3*$^{+/-}$ and *EphA3*$^{-/-}$ embryonic lungs at E15.5 shows a statistically significant difference between *EphA3*$^{+/+}$ and *EphA3*$^{-/-}$ only in epithelial Nkx2-1 expression. Results are mean±s.d. N=3-4 *P < 0,05 (Student's t-test). (C) Constitutive loss of *EphA3* does not have gross effect on embryonic lung branching morphogenesis. Representative E-cadherin whole mount images of *EphA3*$^{+/+}$, *EphA3*$^{+/-}$ and *EphA3*$^{-/-}$ embryonic lungs at E11.5 and E15.5. Image acquisition was performed at 8x magnification for E11.5 lungs, and at 2x for E15.5 lungs.

Supplementary figure 1.

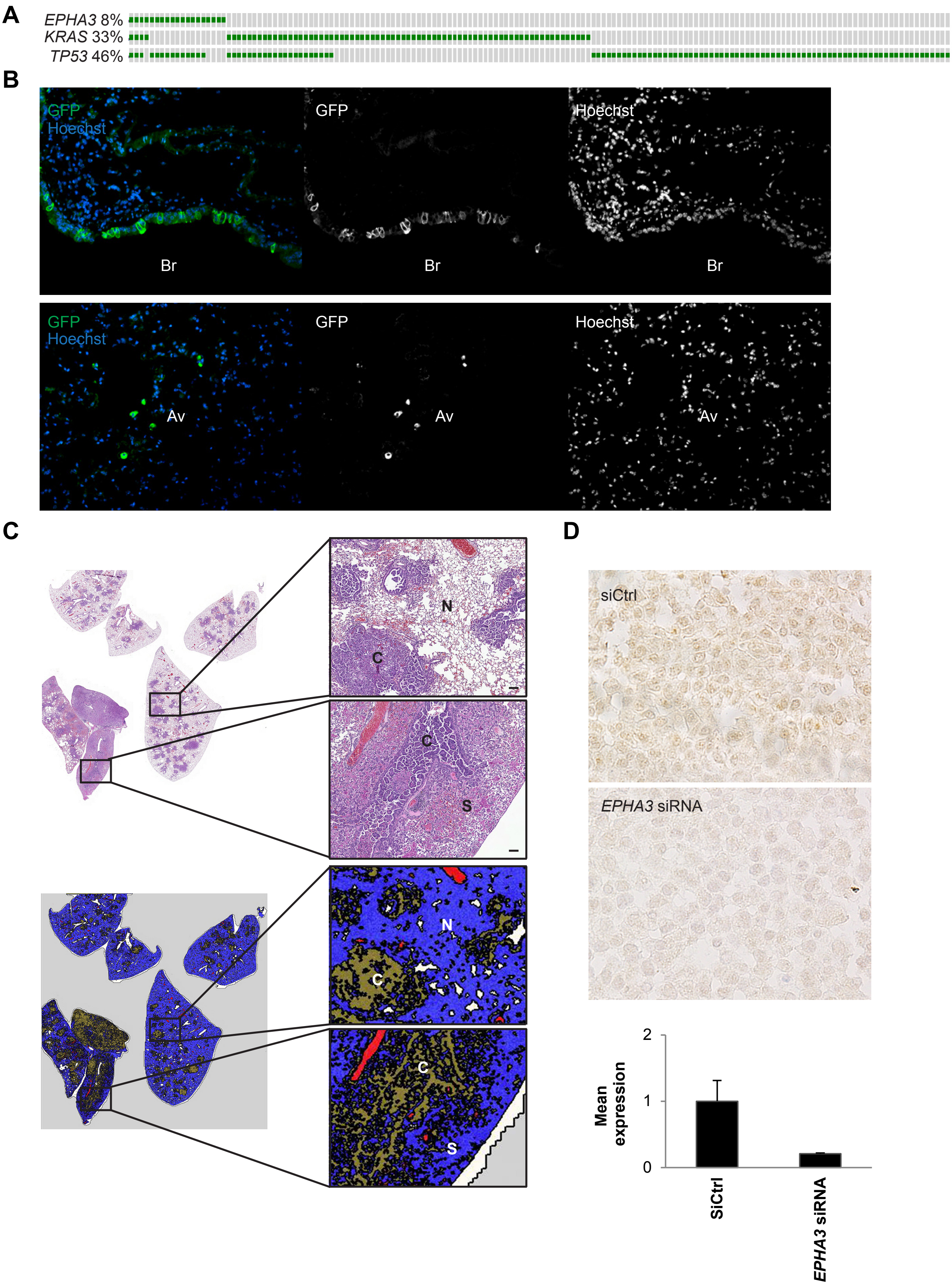

Supplementary figure 2.

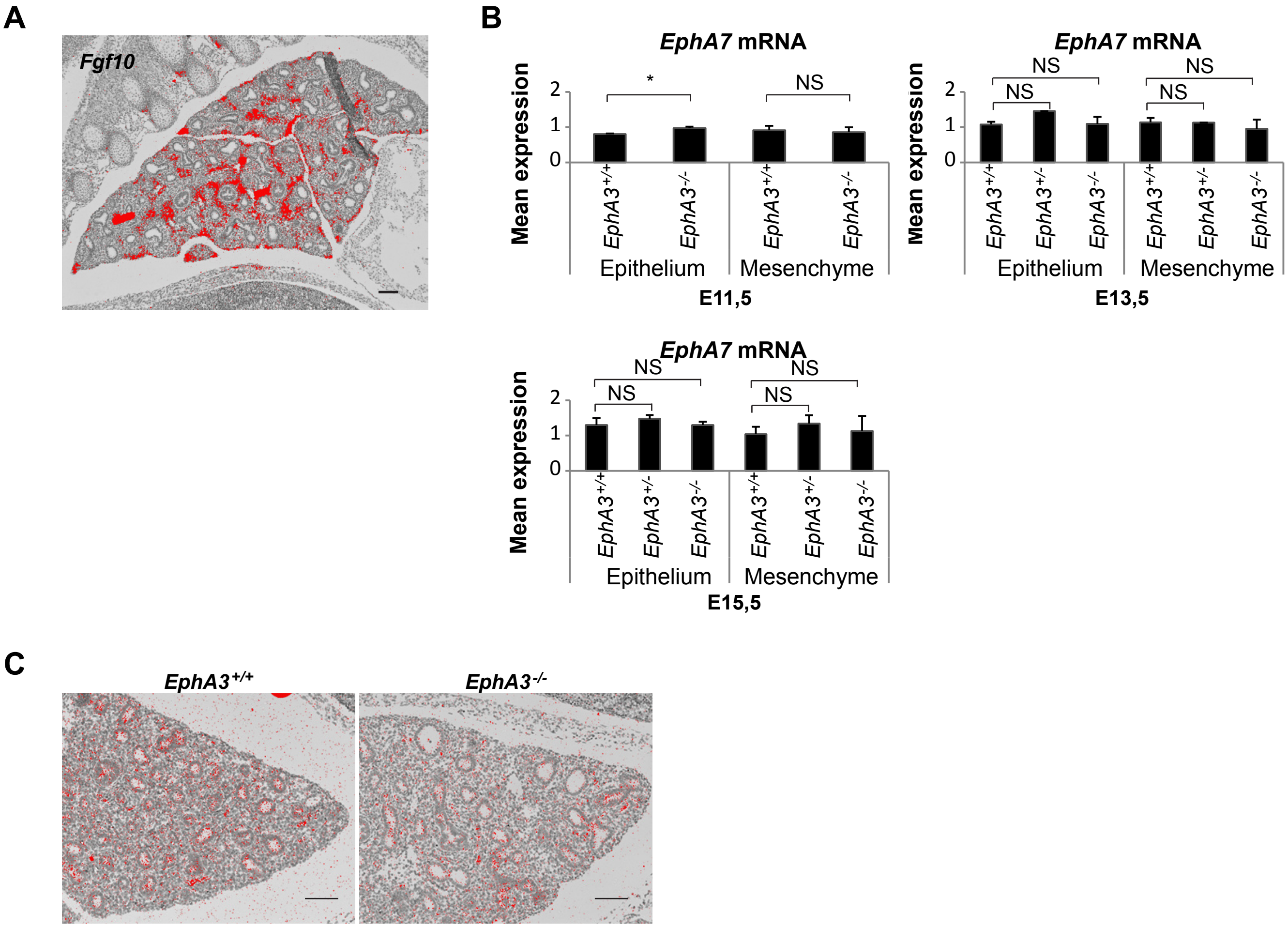

Supplementary figure 3.

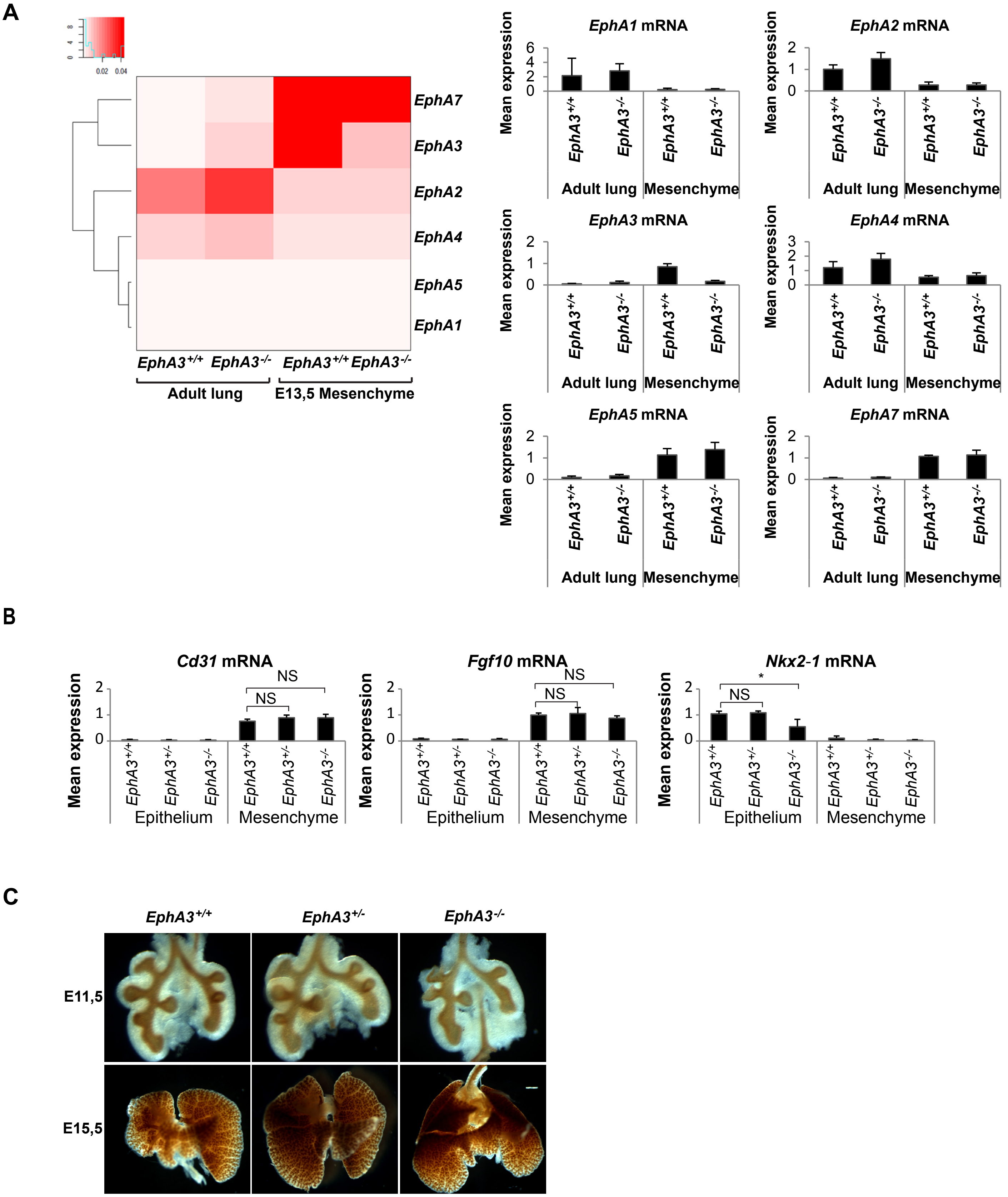

Supplement: Supplementary Material [file supp_8.4.393_DMM019257.pdf]
